# Nitidine chloride suppresses NEDD4 expression in lung cancer cells

**DOI:** 10.18632/aging.202185

**Published:** 2020-12-03

**Authors:** Jing Zhang, Ruoxue Cao, Chaoqun Lian, Tong Cao, Ying Shi, Jia Ma, Peter Wang, Jun Xia

**Affiliations:** 1 Department of Genetics, School of Life Sciences, Bengbu Medical College, Bengbu 233030, Anhui, China; 2 Bengbu Medical College Key Laboratory of Cancer Research and Clinical Laboratory Diagnosis, Bengbu Medical College, Bengbu 233030, Anhui, China; 3 Department of Biochemistry and Molecular Biology, School of Laboratory Medicine, Bengbu Medical College, Bengbu 233030, Anhui, China; 4 Department of Clinical Laboratory, the First Affiliated Hospital of Bengbu Medical College, Bengbu 233004, Anhui, China

**Keywords:** Nitidine chloride, NEDD4, lung cancer, apoptosis, viability

## Abstract

Nitidine chloride (NC) possesses anticancer properties in various types of human malignancies. However, the effects of NC on lung cancer cells have not been elucidated. Moreover, the molecular mechanism of NC-involved antitumor activity is unclear. Therefore, we aimed to determine the biological effect of NC and the underlying molecular insights in lung cancer cells. The antineoplastic function of NC was assessed by MTT assays, Annexin V-FITC/PI apoptosis assay, wound healing analysis, and Transwell chamber migration and invasion assay in lung cancer cells. NEDD4 modulation was evaluated by western blotting assays of lung cancer cells after NC treatments. NEDD4 overexpression and downregulation were employed to validate the critical role of NEDD4 in the NC-mediated tumor suppressive effects. We found that NC suppressed cell viability, migration and invasion, but induced apoptosis in lung cancer cells. Mechanistic exploration revealed that NC exhibited its antitumor effects by reducing NEDD4 expression. Furthermore, our rescue experiments dissected that overexpression of NEDD4 abrogated the NC-mediated antineoplastic effects in lung cancer cells. Consistently, downregulation of NEDD4 enhanced the NC-induced anticancer effects. Thus, NC is a promising antitumor agent in lung cancer, indicating that NC might have potential therapeutic applications in the treatment of lung cancer.

## INTRODUCTION

Lung cancer is one of the most common malignancies worldwide. It is expected that 228,820 new cases of lung cancer will be diagnosed and 112,520 patients will die due to lung cancer in the United States of America in 2020 [1]. Although a reduction in smoking has led to a decline in lung cancer-related deaths, among all types of cancers, lung cancer is the leading cause of cancer-related death in males and females. Deaths due to lung cancer contribute to almost 25% of all cancer deaths in the USA [1]. Treatments for this disease, including surgery, chemotherapy, and immunotherapy, have been improved. However, the 5-year survival rate in lung cancer patients is approximately 19%. Thus, identification of new drugs with fewer side-effects for lung cancer treatments is urgently needed.

NEDD4 (neuronally expressed developmentally downregulated 4) has been characterized as an oncoprotein in carcinogenesis and tumor progression [2]. Several studies have demonstrated that NEDD4 is involved in lung carcinogenesis [3–5]. NEDD4 is highly expressed in 80% of non-small-cell lung carcinoma (NSCLC) tissues, as shown by immunohistochemical assays of tissue microarrays [3]. Upregulation of NEDD4 was correlated with poor prognosis in lung adenocarcinoma [5]. Moreover, NEDD4 suppression attenuated the cell proliferation of NSCLC cells, while overexpression of NEDD4 promoted cell growth in nontransformed lung epithelial cells and lung cancer cells [3]. Another study revealed that NEDD4 negatively regulated PTEN expression, promoting acquired erlotinib resistance in NSCLC [4]. Consistent with this finding, upregulation of NEDD4 is associated with chemoresistance in lung adenocarcinoma [5]. Therefore, inactivation of NEDD4 might be a useful strategy for lung cancer therapy.

Nitidine chloride (NC) has been identified as a phytochemical alkaloid that possessed antifungal, antioxidant and anticancer properties [6]. Accumulated evidence has suggested that NC exerts a tumor suppressive effect via targeting multiple signaling pathways in various human cancers [6]. NC reduced the cell migratory and invasive ability by repressing the c-Src/focal adhesion kinase (FAK) pathway in mammary carcinoma [7]. NC inactivated the signal transducers and activators of transcription 3 (STAT3) signaling pathway and caused attenuation of cell proliferation and angiogenesis in gastric cancer [8]. Similarly, NC suppressed the Janus kinase 1 (JAK1)/STAT3 pathway and caused cell growth inhibition in hepatocellular carcinoma [9]. One study reported that nitidine exhibited cytotoxicity in A549 lung adenocarcinoma cells [10]. Moreover, downregulation of ABCA1 (ATP-binding cassette transporter A1) promoted the cytotoxicity of nitidine in lung cancer cells [10]. However, the effects of NC on lung cancer cells are elusive. Moreover, the underlying molecular mechanisms of NC-mediated antitumor activity need to be explored. Therefore, in the current study, we aimed to elucidate the biological effect and mechanism of NC in lung cancer cells. Our results demonstrated that NC possessed anticancer activity via suppression of NEDD4 in lung cancer.

## RESULTS

### NC suppresses the viability of lung cancer cells

**Figure 1:**
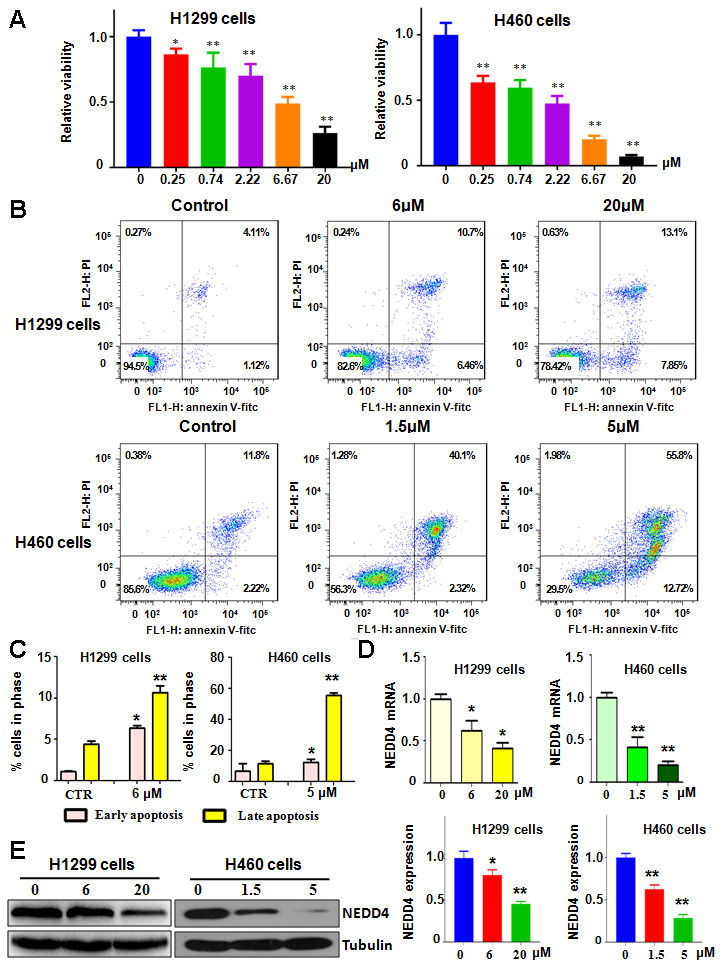
**NC regulates cell viability and apoptosis.** (**A**) The Effects of NC on the viability of lung cancer cells for 72 h were measured using the MTT assay. *P<0.05, **P<0.01 vs the control. (**B**) Cell apoptotic death in lung cancer cells with NC treatment at 72 h was tested by the Annexin V-FITC/PI approach. (**C**) Early and late apoptosis rates were presented for panel B. (**D**) Early NEDD4 mRNA level was detected by RT-PCR in cells with NC treatment for 72 h. (**E**) Left panel: NEDD4 levels were examined by western blotting in lung cancer cells with NC exposure for 72 h. Right panel: Quantitative data are presented for NEDD4 levels. *P<0.05,**P<0.01, vs the control.

To investigate the tumor suppressive function of NC in lung cancer cells, we used an MTT assay to assess the cell viability of H1299 and H460 cells after treatment with different doses of NC for 72 h. We found that NC reduced cell viability in a concentration-dependent manner in lung cancer cells (Figure 1A). Specifically, 6 μM NC resulted in a 50% reduction in cell viability in H1299 cells but an approximately 70% decrease in cell viability in H460 cells, indicating that H460 cells are more sensitive to NC treatment than H1299 cells (Figure 1A). Moreover, 20 μM NC treatment led to 70% and 90% reductions in cell viability in H1299 and H460 cells, respectively (Figure 1A). In the following experiments, we selected 6 μM and 20 μM NC for treating H1299 cells and 1.5 μM and 5 μM NC for H460 cell treatment.

### NC induces apoptotic death of lung cancer cells

To define the effects of NC on lung cancer cells, we assessed the apoptotic death of H1299 and H460 cells after different concentrations of NC for 72 h. Our Annexin V-FITC/PI assay showed that NC exposure induced cell apoptosis in both H1299 and H460 cells (Figure 1B). The results revealed that 6 μM and 20 μM NC increased cell apoptosis from 5.23% in the control group to 17.1% and 20.9% in H1299 cells, respectively (Figure 1B). The early apoptosis rate was increased from 1.12% to 6.46% and the late apoptosis rate was elevated from 4.11% to 10.7% in H1299 cells after 6 μM NC treatment (Figure 1C). Similarly, 1.5 μM and 5 μM NC triggered cell apoptosis from 14% in the control group to 42.4% and 68.5% in H460 cells, respectively (Figure 1B). The early apoptosis rate was increased from 2.22% to 12.72% and the late apoptosis rate was elevated from 11.8% to 55.8% in H460 cells after 5 μM NC treatment (Figure 1C). Altogether, NC induced cell apoptosis in lung cancer cells.

### NC reduced NEDD4 expression

NEDD4 functions as a key oncoprotein in carcinogenesis. We explored whether NC performed cell viability inhibition via downregulation of NEDD4 in lung cancer cells. To achieve this aim, we used RT-PCR and western blotting analysis to assess the expression of NEDD4 in H1299 and H460 cells after NC treatments. Our data showed that NC suppressed the expression of NEDD4 at both mRNA and protein levels in both H1299 and H460 cells (Figure 1D, 1E). This finding indicates that NEDD4 inhibition by NC could be a reason for the NC-mediated suppression of cell viability.

### NC attenuates cell migration and invasion

**Figure 2:**
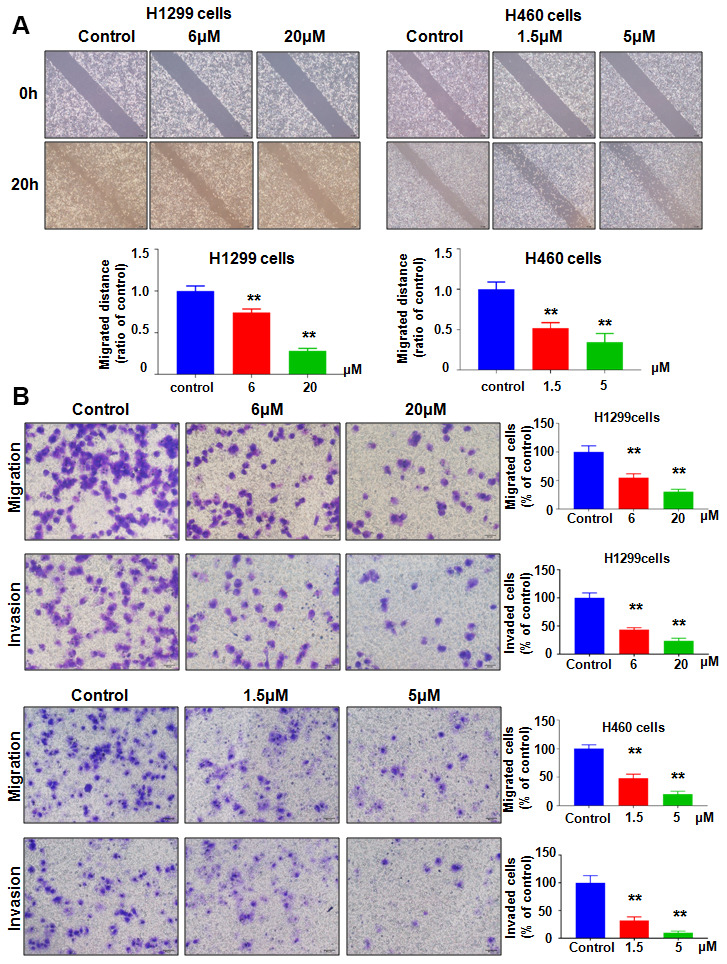
**NC regulates cell migration and invasion.** (**A**) Top panel: A wound healing assay was used to measure the motility of lung cancer cells. Bottom panel: Quantitative data are presented for wound healing. **P<0.01 vs the control. (**B**) Left panel: Migration and invasion were measured in lung cancer cells treated with NC for 24 h. Right panel: Quantitative data are presented for motility. **P<0.01 vs the control.

NEDD4 governs cell motility in tumor cells. Therefore, we tested whether NC inhibited NEDD4 expression and led to suppression of migration and invasion of lung cancer cells. Our wound healing assay showed that NC exposure resulted in retardation of wound closure in both H1299 and H460 cells (Figure 2A). Transwell chamber migration and invasion assays dissected that NC exposure attenuated the cell migratory and invasive activity of H1299 and H460 cells (Figure 2B). Altogether, the mobility of lung cancer cells was decreased with NC treatment.

### Overexpression of NEDD4 neutralized the anticancer effects of NC

**Figure 3:**
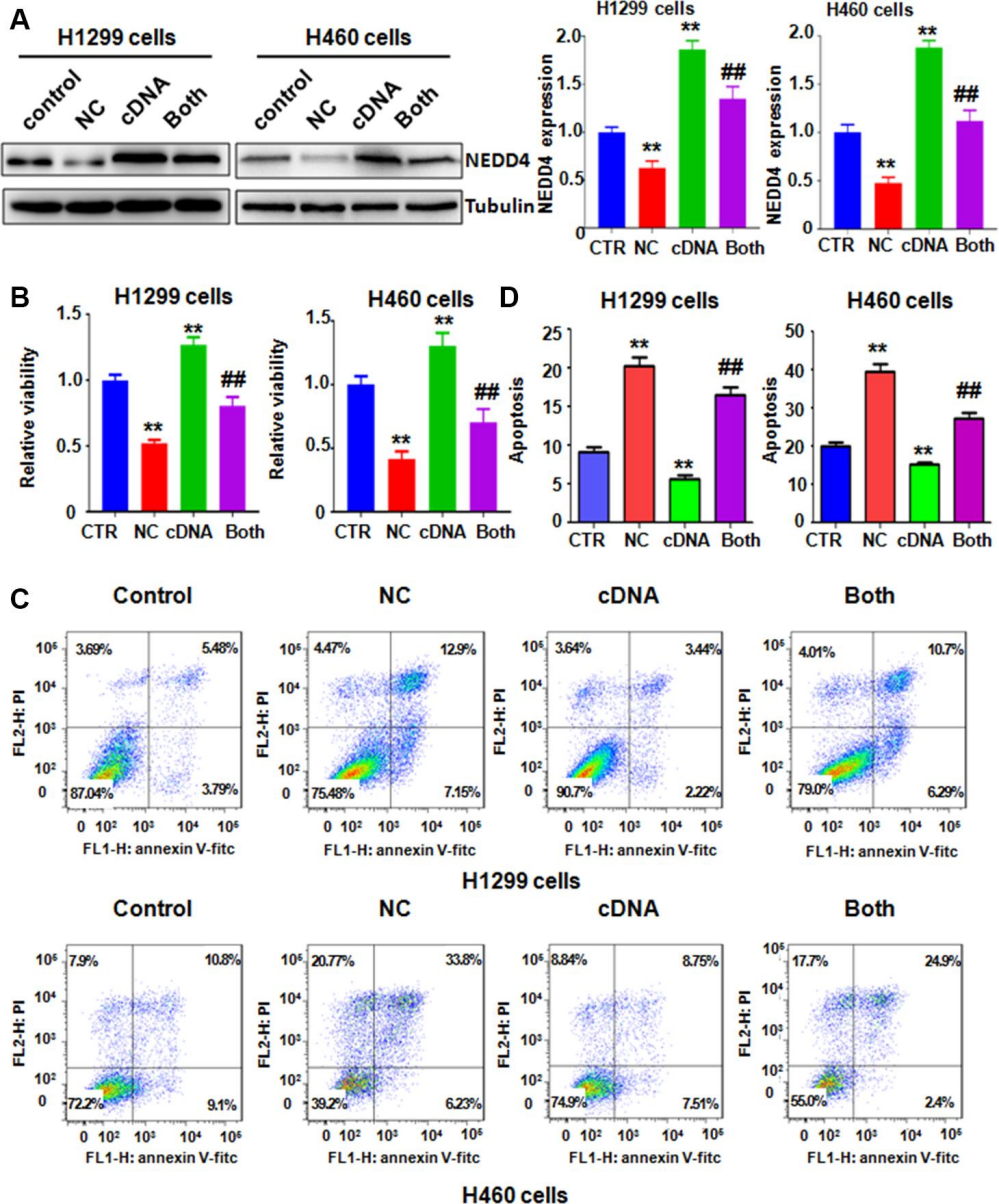
**NEDD4 overexpression rescues NC-mediated cell growth suppression and apoptosis.** (**A**) Left panel: NEDD4 expression was tested by immunoblotting in lung cancer cells with NEDD4 plasmid transfection plus NC treatment. Right panel: Quantitative data are shown for NEDD4 levels. **P<0.01, vs the control; ^##^p<0.01, vs NC only or NEDD4 plasmid transfection only. CTR: control; NC: Nitidine chloride; cDNA: NEDD4 cDNA vector; Both: NEDD4 cDNA plus NC. (**B**) The MTT assay was carried out to test the viability of lung cancer cells after NEDD4 overexpression and NC treatment. (**C**) Apoptosis of lung cancer cells was examined by flow cytometry after the combination treatment. (**D**). Apoptosis rates were presented for panel **C**.

**Figure 4:**
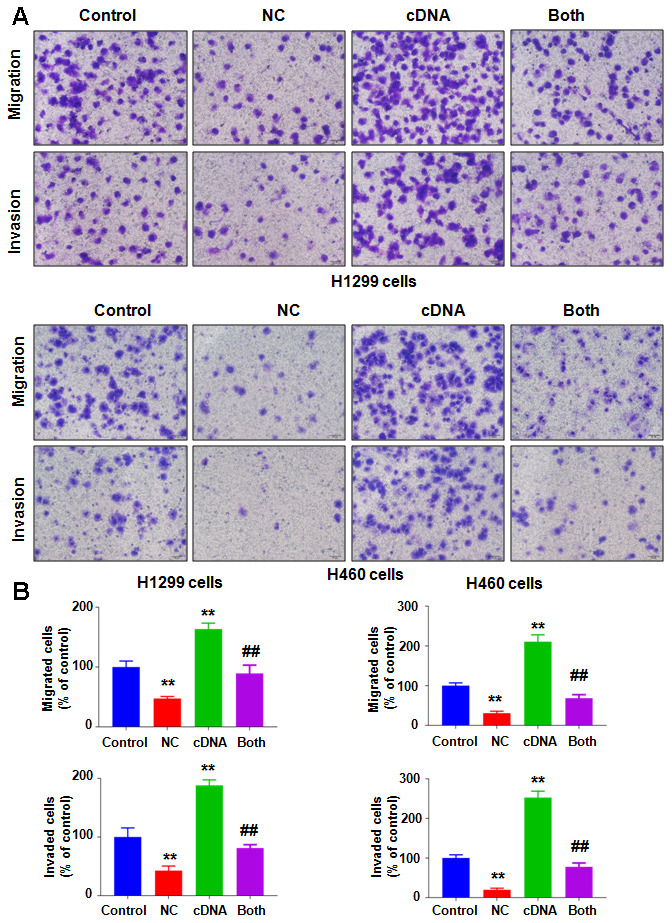
**Overexpression of NEDD4 attenuates NC-involved inhibition of cell motility.** (**A**) Cell migration and invasion were evaluated in lung cancer cells after the combination treatments. (**B**) Quantitative data are shown for panel **A**. **P<0.01, vs the control; ^##^p<0.01, vs NC only or NEDD4 plasmid transfection only.

To evaluate whether the antitumor effects of NC are mediated via regulation of NEDD4, we transfected a cDNA plasmid into lung cancer cells to increase the NEDD4 level and detected whether upregulation of NEDD4 could reverse the anticancer effect of NC on lung cancer cells. Our western blotting data showed that NEDD4 was highly expressed in H1299 and H460 cells after NEDD4 cDNA plasmid transfection (Figure 3A). Strikingly, NEDD4 cDNA transfection rescued the reduction of NEDD4 induced by NC treatment in both H1299 and H460 cells (Figure 3A). Next, we assessed whether increased NEDD4 abolished the NC-triggered suppression of cell viability in lung cancer cells. Our MTT data demonstrated that NEDD4 overexpression promoted cell viability in H1299 and H460 lung cancer cells (Figure 3B). Moreover, overexpression of NEDD4 neutralized the reduction in cell viability induced by NC exposure in lung cancer cells (Figure 3B). In support of these data, we observed that NEDD4 upregulation reduced the apoptotic death of lung cancer cells (Figure 3C, 3D). Importantly, NEDD4 upregulation the abrogated NC-triggered induction of cell apoptosis in H1299 and H460 lung cancer cells (Figure 3C, 3D). The migration of lung cancer cells after NEDD4 cDNA transfection plus NC exposure was also evaluated by Transwell chamber migration assay. NEDD4 upregulation facilitated the migratory activity of H1299 and H460 cells (Figure 4A, 4B). Notably, upregulation of NEDD4 abolished the NC-induced retardation of cell migration in lung cancer cells (Figure 4A, 4B). Consistently, NEDD4 overexpression by cDNA transfection enhanced the cell invasive ability of H1299 and H460 cells (Figure 4A, 4B). Overexpression of NEDD4 reversed the NC-involved reduction in cell invasion in lung cancer (Figure 4A, 4B). Thus, NC conducted antitumor effects in lung cancer cells in part via attenuation of NEDD4 expression.

### Downregulation of NEDD4 promotes the antitumor activity of NC

**Figure 5:**
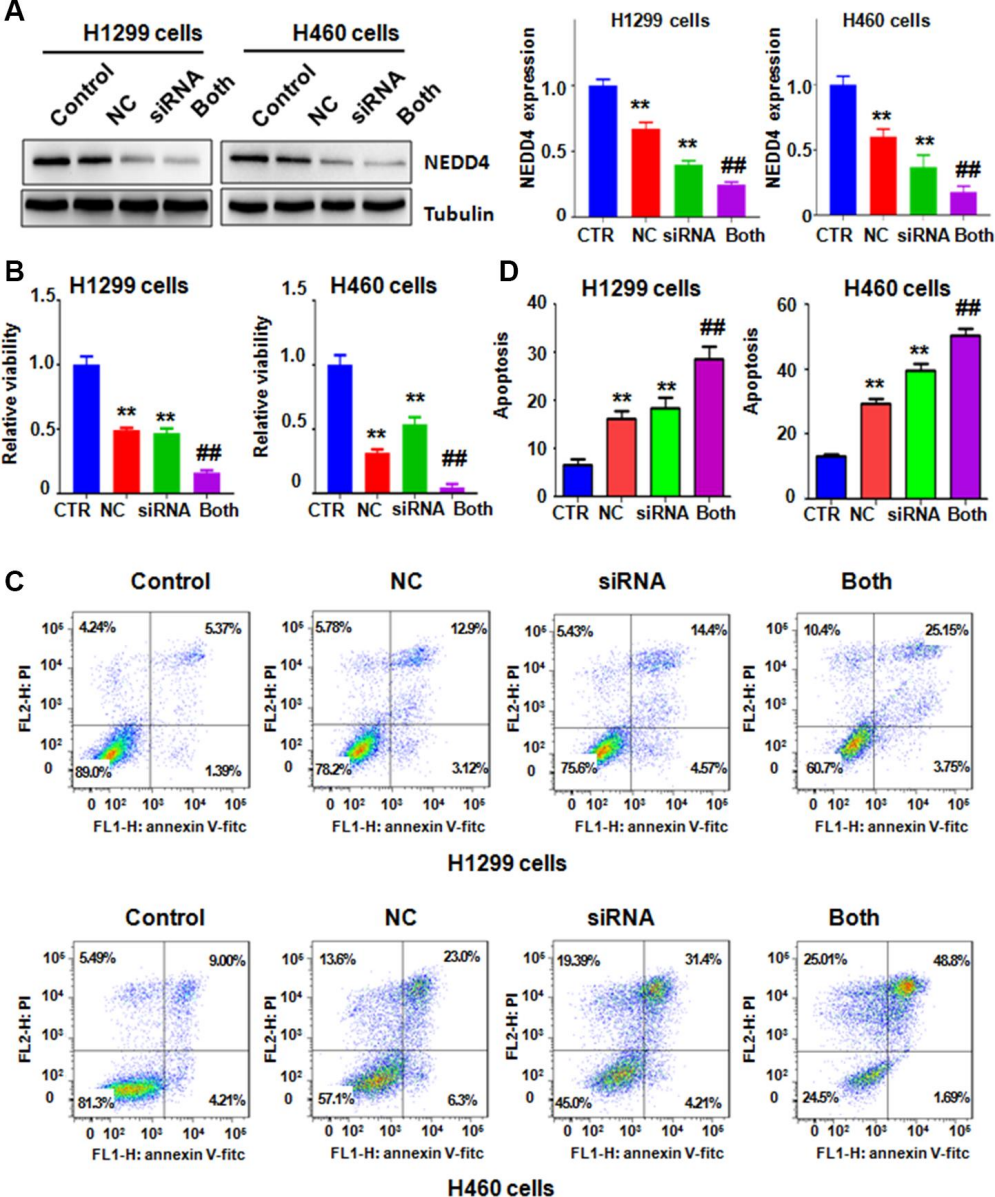
**NEDD4 downregulation promotes NC-mediated cell growth suppression and apoptosis.** (**A**) Left panel: NEDD4 expression was tested by immunoblotting in lung cancer cells with different treatments. Right panel: Quantitative data from western blotting. **P<0.01, vs the control; ^##^p<0.01, vs NC only or NEDD4 siRNA transfection only. CTR: control; NC: Nitidine chloride; siRNA: NEDD4 siRNA; Both: NEDD4 siRNA plus NC. (**B**) The MTT assay measured the viability of lung cancer cells after the combination treatments. (**C**) Cell apoptosis in lung cancer cells was examined by flow cytometry after the combination treatments. (**D**) Apoptosis rates were presented for panel **C**.

**Figure 6:**
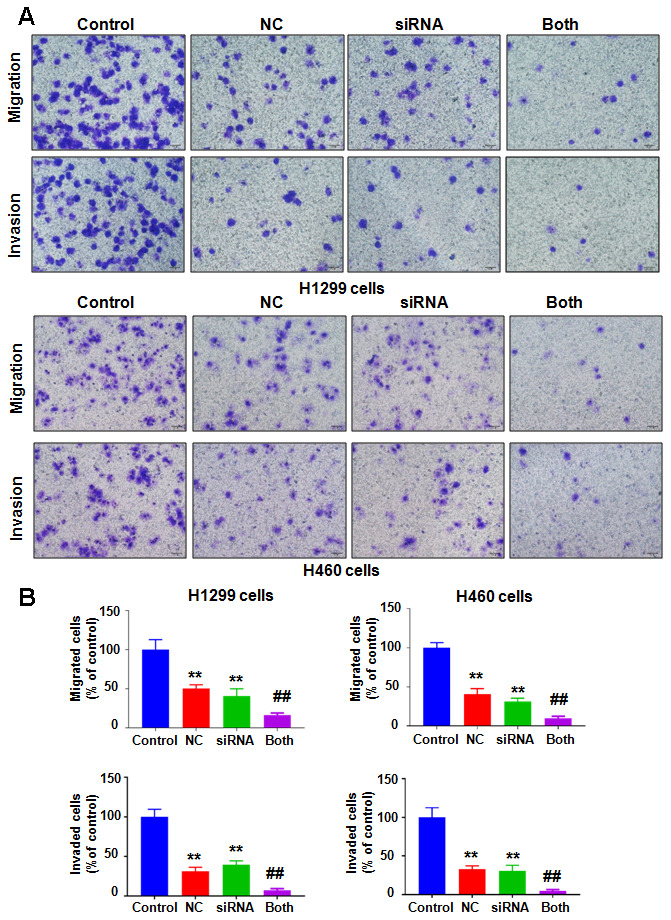
**Downregulation of NEDD4 enhances NC-involved inhibition of cell motility.** (**A**) Cell migration and invasion were evaluated in lung cancer cells after combination treatments. NC: Nitidine chloride; siRNA: NEDD4 siRNA; Both: NEDD4 siRNA plus NC. (**B**) Quantitative data are shown for motility. **P<0.01, vs the control; ^##^p<0.01, vs NC only or NEDD4 siRNA transfection only.

To define the potential role of NEDD4 in NC-involved antitumor activity in lung cancer, we decreased NEDD4 expression using NEDD4 siRNA transfection and cotreated the H1299 and H460 cells with NC. Our immunoblotting data demonstrated that siRNA treatment remarkably reduced the expression of NEDD4 in H1299 and H460 cells (Figure 5A). NC in combination with NEDD4 siRNA transfection reduced the NEDD4 level to a lower level than that with single NC exposure or NEDD4 siRNA treatment only (Figure 5A). MTT data revealed that NEDD4 downregulation reduced cell viability in H1299 and H460 cells (Figure 5B). The NC and NEDD4 siRNA combinations led to a greater reduction in cell viability of lung cancer cells than that after single treatment (Figure 5B). Similarly, NEDD4 suppression by siRNA transfection stimulated the apoptosis of H1299 and H460 lung cancer cells (Figure 5C, 5D). The combination of NC and NEDD4 downregulation led to enhanced cell apoptosis compared with single treatment (Figure 5C, 5D). Finally, NEDD4 inhibition attenuated the migratory and invasive abilities ofH1299 and H460 lung cancer cells (Figure 6A, 6B). Consistently, combination treatments of the lung cancer cells led to a higher suppression of cell migration and invasion than NC treatment alone or NEDD4 downregulation alone (Figure 6A, 6B).

## DISCUSSION

Evidence has revealed that NEDD4 plays a critical role in the development and progression of human cancer, including NSCLC [2, 3]. One group reported that NEDD4 knockdown reduced proliferation, migration and invasion in A549 lung cancer cells via targeting phosphatase and tensin homolog (PTEN) and the phosphatidylinositol 3-kinase (PI3K)/Akt pathway [5]. Moreover, this group found that NEDD4 downregulation enhanced the chemosensitivity of A549 cells to cisplatin and paclitaxel [5]. Another group observed that knockdown of NEDD4 decreased EGF-induced cell migration via suppression of cathepsin B lysosomal secretion in NSCLC cells [11]. Because NEDD4 acts as a tumor promoter in NSCLC, inactivation of NEDD4 might be a potential treatment for NSCLC patients.

NC blocked the Akt pathway and retarded cell metastasis of renal cancer [12]. Specifically, NC reduced the level of phosphorylation of Akt and downregulated matrix metalloproteinase-2 (MMP-2) and MMP-9, leading to antimetastatic effects on renal cancer cells [12]. NC repressed cell proliferation and stimulated apoptosis via inhibition of the extracellular signal regulated kinases (ERK) pathway in renal cancer [13]. One study showed that NC reduced cell viability and promoted apoptosis through enhancement of p53 in nasopharyngeal carcinoma cells [14]. Another study revealed that NC treatments caused apoptosis and cell cycle arrest at G2/M phase, and led to an enhanced efficacy of doxorubicin on the growth suppression of breast cancer [15]. Similarly, NC exhibited anticancer activity and a synergistic effect with doxorubicin by targeting the Akt and Fas signaling pathways in ovarian cancer cells [16, 17]. NC inactivated multiple signaling pathways, including the STAT3, ERK and SHH pathways, and modulated several key genes in hepatic cancer cells: cell cycle related genes (Cyclin D1, CDK4, p53, p21), cell apoptosis related genes (Bcl-2 and Bax), and angiogenesis-related genes (vascular endothelial growth factor-A; VEGF-A and VEGF receptor-2; VEGFR2) [18, 19]. In addition, NC performed antitumor activities via inactivation of the ERK pathway in colorectal cancer and ovarian cancer [20, 21]. Intriguingly, NC attenuated epithelial to mesenchymal transition (EMT) via regulation of Akt and glycogen synthase kinase-3β (GSK-3β)/Snail, leading to suppression of migration and invasion in osteosarcoma cells [22]. Moreover, NC inhibited EMT and cancer stem cell properties in breast cancer, glioma and colon cancer via targeting the hedgehog, ERK and Akt pathways [23]. NC exhibited inhibition of the malignant behavior via inactivation of the PI3K/Akt/mTOR pathway in glioblastoma cells [24]. Our previous studies showed that NC downregulated Yes-associated protein (YAP) in prostate cancer cells and reduced SIN1 in osteosarcoma cells [25, 26]. Here, we reported that NC suppressed the expression of NEDD4 in lung cancer cells. Thus, NC might be a promising agent for treating human cancers via inhibition of NEDD4.

Several groups have reported that multiple natural compounds inhibit the expression of NEDD4 in a variety of human cancers [27–30]. Indole-3-carbinol had antiproliferative effects via binding with NEDD4 and subsequently impairing PTEN degradation in melanoma cells. [28] I3C analogs disrupted cell proliferation via suppression of NEDD4 ligase activity in melanoma cells [27]. Paeoniflorin downregulated the expression of NEDD4 and led to antitumor effects on nasopharyngeal carcinoma cells [29]. Diosgenin exerted its anticancer function via downregulation of NEDD4 in prostate cancer cells [30]. In the current study, we used a cell line system to define the biological function of NC in vitro via modulation of NEDD4 in NSCLC. Whether NC attenuated tumor growth in mice via suppression of NEDD4 needs to be determined, which could be helpful for the potential use of NC in clinical trials in the future. In summary, NC might be a promising inhibitor of NEDD4 in NSCLC.

## MATERIALS AND METHODS

### Cell culture

H1299 and H460 lung cancer cells were purchased from the Chinese Academy of Science Cell Bank (Shanghai, China). H1299 cells and H460 cells were cultured in RPMI-1640 medium with 10% FBS and 1% penicillin/streptomycin. Cells were incubated in a humidified environment with 5% CO_2_ at 37° C.

### Cell viability assay

Cells were seeded onto 96-well plates for 12 h and then exposed to various doses of NC or transfected with NEDD4 siRNA or cDNA plasmid for 72 h. Cell viability was detected by MTT assays.

### Cell apoptosis assay

Cells were seeded in 6-well plates for 12 h and then treated with different doses of NC for 72 h. Cell apoptotic death was detected via measuring cells stained with both FITC-Annexin V and propidium iodide (PI) by flow cytometry. FlowJo software was used to calculate the apoptosis rate.

### Cell wound healing assay

Cells were incubated in 6-well plates until cells became more than 90% confluent. A yellow sterile pipette tip was used to scratch a wound, and cells in the supernatant were removed by washing. The cells were exposed to various doses of NC for 20 h. The wound fields were captured by a microscope when cells were treated with NC for 20 h.

### Transwell migration and invasion assay

Cell migration and invasion were assessed by 24-well plate Transwell chambers as described previously [31]. The migration and invasion were assessed in cells after NC exposure as described before [26]. Five independent fields were captured by a microscope, and the stained cell numbers were counted.

### Transfection

Cells were transfected with NEDD4 cDNA plasmid or NEDD4 siRNA (GenePharma Company, Shanghai, China) by Lipofectamine 2000 (Invitrogen, Carlsbad, CA, USA) as described before [26]. The NEDD4 cDNA plasmid was purchased from Addgene Company.

### Real-time RT-PCR analysis

The total RNA was extracted using TRIzol reagent. The mRNA level of NEDD4 was examined using the SYBR green RT-PCR approach as described previously [32].

### Western blotting analysis

Cells were lysed, and proteins were extracted. Then, protein amounts were measured by BCA assay. Protein was separated by SDS-PAGF and transferred onto PVDF membranes. Anti-NEDD4 antibody was purchased from CST Company (Danvers, MA, USA). Western blotting was performed as described previously [26].

### Statistical analysis

Data were evaluated by GraphPad Prism 6.0 software (CA, USA). ANOVA (analysis of variance) was used to analyze differences in multiple groups. P<0.05 was considered the significance threshold.
